# Factor structure and psychometric properties of the Connor-Davidson resilience scale among Brazilian adult patients: Estrutura fatorial e propriedades psicométricas da Escala de Resiliência de Connor-Davidson para pacientes brasileiros adultos

**DOI:** 10.1590/1516-3180.2015.02290512

**Published:** 2016-05-13

**Authors:** João Paulo Consentino Solano, Eduardo Sawaya Botelho Bracher, Alexandre Faisal-Cury, Hazem Adel Ashmawi, Maria José Carvalho Carmona, Francisco Lotufo, Joaquim Edson Vieira

**Affiliations:** I MD, MSc. Doctoral Student and Attending Psychiatrist in the Pain Control Group, Department of Anesthesiology, Faculdade de Medicina da Universidade de São Paulo (FMUSP), São Paulo, SP, Brazil.; II MD, PhD. Director, Axis Clínica de Coluna, São Paulo, SP, Brazil.; III MD, PhD. Medical Researcher, Department of Preventive Medicine, Faculdade de Medicina da Universidade de São Paulo (FMUSP), São Paulo, SP, Brazil.; IV MD, PhD. Head of Pain Control Team, Department of Anesthesiology, Faculdade de Medicina da Universidade de São Paulo (FMUSP), São Paulo, SP, Brazil.; V MD, PhD. Associate Professor, Department of Anesthesiology, Faculdade de Medicina da Universidade de São Paulo (FMUSP), São Paulo, SP, Brazil.; VI MD, PhD. Head of Department of Psychiatry, Faculdade de Medicina da Universidade de São Paulo (FMUSP), São Paulo, SP, Brazil.; VII MD, PhD. Associate Professor, Department of Anesthesiology, Faculdade de Medicina da Universidade de São Paulo (FMUSP), São Paulo, SP, Brazil.

**Keywords:** Resilience, psychological, Cross-cultural comparison, Validation studies [publication type], Psychometrics, Questionnaires, Resiliência psicológica, Comparação transcultural, Estudos de validação, Psicometria, Questionários

## Abstract

**CONTEXT AND OBJECTIVE:**

Personal resilience is associated with several mental health outcomes. The Connor-Davidson resilience scale (CD-RISC) is a widely used self-report measurement of resilience. This study aimed to investigate the reliability and validity of a Brazilian Portuguese version of the CD-RISC.

**DESIGN AND SETTING:**

Cross-sectional validation study carried out in the outpatient clinics of a public university hospital.

**METHODS:**

The cross-cultural adaptation followed established guidelines and involved interviews with 65 adults in psychiatric and non-psychiatric outpatient clinics at a teaching hospital. Validation was assessed through concurrent application of the Lipp Brazilian Stress Symptom Inventory (ISSL), Self-Report Questionnaire (SRQ), Sheehan Disability Scales (SDS) and Chronic Pain Grade (CPG) to 575 patients at the same setting. Temporal stability was verified through a second application to 123 participants.

**RESULTS:**

Factor analysis identified four factors, named tenacity, adaptability-tolerance, reliance on support from outside and intuition. The alpha coefficient of 0.93 and intraclass correlation coefficient of 0.84 indicated good internal consistency and temporal stability. Significant correlations between this version of the CD-RISC and the ISSL, SRQ, SDS and CPG were noted. The patients at the outpatient clinic for borderline personality had resilience scores that were significantly lower than those of the patients at the general anxiety or post-traumatic stress outpatient clinics.

**CONCLUSION:**

This Brazilian Portuguese version of the Connor-Davidson resilience scale exhibited adequate reliability and validity among a sample of Brazilian adult patients.

## RESUMO

**CONTEXTO E OBJETIVO:**

A resiliência pessoal está associada a diversos desfechos em saúde mental. A escala de resiliência de Connor-Davidson (CD-RISC) vem sendo amplamente empregada como uma medida autorrelatada de resiliência. Este estudo teve por objetivo verificar a confiabilidade e a validade de uma versão da CD-RISC para o português no contexto cultural brasileiro.

**DESENHO E LOCAL:**

Estudo transversal de validação conduzido nos ambulatórios de hospital público universitário.

**MÉTODOS:**

De acordo com diretrizes bem conhecidas, a adaptação cultural foi feita com 65 adultos entrevistados em ambulatórios psiquiátricos e não psiquiátricos de um hospital de ensino. A validação se deu pela aplicação concorrente do Inventário de Stress para Adultos de Lipp (ISSL), Questionário de Autorrelato de Sintomas (SRQ), Escalas de Incapacidade de Sheehan (SDS) e Escala Graduada de Dor Crônica (CPG) a 575 pacientes do mesmo hospital. A estabilidade temporal foi verificada numa segunda aplicação a 123 participantes.

**RESULTADOS:**

A análise fatorial identificou quatro fatores, nomeados como tenacidade, adaptabilidade-tolerância, amparo e intuição. Um coeficiente alfa de 0,93 e um coeficiente de correlação intraclasse de 0,84 indicaram adequadas consistência interna e estabilidade temporal. Correlações significativas entre esta versão da CD-RISC e o ISSL, SRQ, SDS e CPG foram identificadas. Os pacientes do ambulatório para personalidade *borderline* tiveram escores de resiliência significativamente mais baixos que os pacientes dos ambulatórios geral de ansiedade ou de estresse pós-traumático.

**CONCLUSÃO:**

A presente versão em português da escala de resiliência de Connor-Davidson apresentou confiabilidade e validade adequadas numa amostra de pacientes brasileiros adultos.

## INTRODUCTION

Resilience is a construct associated with the ability to adapt when challenged by stressors or adversities, or to strive despite the toughness of circumstances that are experienced.^1^^,^^2^ The concept is rooted in other fields of science (physics, engineering and dentistry) where it relates to the resistance of materials.^3^ Resilient materials are flexibly capable of non-permanent deformation, a property that allows them to accumulate energy and thus avoid breakage under mechanical stress. Likewise, resilient individuals (or communities) are able to adjust rapidly to the adversities of life, thus remaining on the path of wellness. Since this allegorical translation of the term resilience as a psychological construct was first made, some features usually displayed by resilient people have been reported: realistic optimism, highly positive emotionality, sense of purpose in life, an internal framework of beliefs about right and wrong, spirituality, use of active coping strategies such as problem solving and planning, ability to find meaning even in traumatic experiences, and the tendency to perceive stressful events in less threatening ways and to reframe adverse experiences in a more positive light.^4^^,^^5^

Although seminal authors in the field of psychological resilience have mainly investigated children under unfavorable conditions (e.g. poverty or chronic maltreatment), more recent papers have also focused on (a) traumatic experiences of both children and adults and their outcomes and (b) the interrelationships between resilience and chronic stressors.^6^^,^^7^ Among chronic stressors, attention has been paid to people enduring chronic illnesses and ailments.^8^^,^^9^^,^^10^^,^^11^ In a country like Brazil where the population is rapidly growing older, the resilience of people facing chronic diseases and associated limitations does matter.^12^

There has been notable interest in developing assessment tools for measuring individual resilience. In a review, Ahern et al. identified six measurements of resilience.^13^ Five years later, a review by Windle et al. analyzed 15 measurements.^14^ In the latter, instruments were ranked according to several of their attributes (consistency, length of fit, etc.), and the Connor-Davidson Resilience Scale (CD-RISC) was one of the top-ranked instruments.^15^

## OBJECTIVE

The objective of the present study was to investigate the reliability, validity and factor structure of a culturally adapted Brazilian Portuguese version of the Connor-Davidson Resilience Scale, in a sample of adult outpatients.

## METHODS

The protocol for this study was approved by the Institutional Review Board of the teaching hospital of a public university medical school. Cultural adaptation procedures were conducted in accordance with the guidelines proposed by Beaton et al. and Guillemin.^16^^,^^17^

### Connor-Davidson Resilience Scale (CD-RISC)

The CD-RISC^15^ is a 25-item questionnaire for evaluating individual resilience. Its reliability and validity have been studied in populations in North America,^15^^,^^18^ Europe,^19^^,^^20^^,^^21^ Africa^22^ and Asia.^23^^,^^24^^,^^25^^,^^26^ Respondents rate items on a scale from 0 (“not true at all”) to 4 (“true nearly all the time”). The original study on the development of the CD-RISC in the general population and in patient samples provided support for the internal consistency, test-retest reliability and validity of this scale.

### Participants

For the cross-cultural adaptation phase, 65 adult patients (18 years or older) were approached in the waiting rooms of either the general outpatient clinic for anxiety disorders or the outpatient clinic for pre-anesthetic consultations for elective surgeries of the medical school’s teaching hospital. For the validation phase, patients in the waiting rooms of the outpatient clinics for borderline personality disorder, post-traumatic stress disorder and chronic pain, and adult companions of pre-anesthetic consultation patients, were also approached. If these individuals presented reading and hearing disabilities or cognitive impairment, the interview was halted and the individual was excluded from the study (exclusion criteria of the study protocol). Psychiatric patients were interviewed only after the consultant psychiatrist had stated that the patient’s diagnosis was among those pre-specified in the inclusion criteria of the study protocol (borderline personality, post-traumatic stress disorder or other anxiety disorder). All the participants signed an informed consent statement before the interview was started.

### Cross-cultural adaptation phase

**Figure 1.**
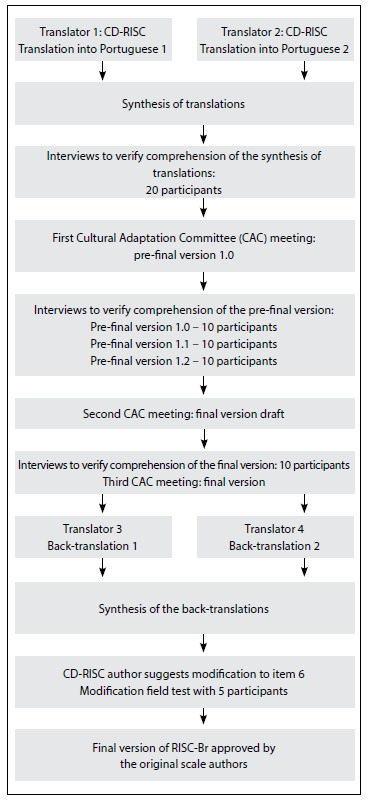
Cross-cultural adaptation process for the Brazilian version of the Connor-Davidson Resilience Scale.

For the cultural adaptation phase, two specialists in English-Portuguese translations (of whom one was a specialist in adult literacy in Portuguese) independently prepared Portuguese versions of the CD-RISC. A synthesis between the two versions was obtained through consensus agreement between the translators. A cultural adaptation committee (CAC) was then created, including both of the specialists in English-Portuguese translations, a psychologist, a psychiatrist, an epidemiologist and a physical medicine and rehabilitation doctor. The comprehension of the Portuguese version was verified through interviews with subjects within the target population, during which the respondents were asked about their understanding of each question and invited to offer suggestions for words or expressions that might clarify their meaning. At three successive meetings, the cultural adaptation committee discussed the ongoing results from the interviews and suggested changes to the Portuguese version, with the aim of improving comprehension while maintaining equivalence with the original instrument. The final version was defined after 60 patients had been interviewed. Two independent back-translations of the final version were made by native English-speaking professional translators, and a synthesis was agreed upon through reaching a consensus. The authors of the original instrument were contacted, and agreed that conceptual equivalence had been maintained between the back-translation and the original instrument. One of the authors proposed a minor alteration to item 6. After this item had been altered, five additional interviews were conducted to test the adequacy of the modification. The final version was then named the Connor-Davidson Resilience Scale Brazil (RISC-Br). Figure 1 presents a flowchart of the cross-cultural adaptation process.

### Validation phase

The validation assessments used included concurrent application of the RISC-Br, the Lipp Brazilian Stress Symptom Inventory,^27^ the Brazilian version of the Self-Report Questionnaire,^28^ the Sheehan Disability Scale^29^ and the Brazilian version of the Chronic Pain Grade^30^ to 575 participants who were attending the hospital’s outpatient clinics. We expected to find an inverse relationship between resilience and distressing symptoms as measured using the Brazilian Lipp Stress Symptom Inventory, Self-Report Questionnaire and the pain intensity subscale of the Chronic Pain Grade, as well as between resilience and the self-reported negative impact of such symptoms as measured using the Sheehan Disability Scale and the two subscales of activity limitation due to pain in the Chronic Pain Grade. In other words, lower resilience was expected to be associated with higher scores in these instruments. We also expected that patients enduring chronic pain would probably display greater resilience, and that borderline patients would have the lowest resilience scores. Test-retest reliability was studied by means of a second interview, which was conducted between 7 and 14 days after the first encounter.

### Data analysis

The demographic and clinical characteristics of the sample were established through descriptive analysis. Exploratory factor analysis was performed on the data from the validation phase (n = 575). In accordance with Kaiser’s rule, principal components with eigenvalues greater than 1.0 were selected for oblique (direct oblimin) rotation. Oblique rotation is preferable when the construct under exploration is expected to have dimensions (factors) that relate to each other.^31^ Exploratory factor analysis yielded four factors accounting for more than 55% of the variance of the scale. Cronbach’s alpha coefficient was used to assess internal consistency for each factor and for the whole scale. Intraclass correlation coefficients were calculated in order to assess the test-retest reliability using a subsample of the interviewees who were contacted on a second occasion (n = 123). Spearman coefficient correlations were used to assess construct validity. Thirteen items were used as comparison criteria: the six subscales of the Brazilian Lipp Stress Symptom Inventory, the Self-Report Questionnaire, the three subscales of the Sheehan Disability Scale and three subscales of the Chronic Pain Grade. Although not pertaining to the formal objective of the study, the mean resilience scores from the six subsamples of the validation phase were tested for differences by means of analysis of variance (ANOVA).

## RESULTS

### Descriptive statistics

The majority of the participants were women (428; 74%), with an average age of 44 years (range: 18-81) and 10 years of formal schooling. The participants were predominantly married (56%) and of socioeconomic levels B or C (92%), on a scale from A to E. Table 1 shows the sample distribution according to sociodemographic characteristics.

**Table 1.**
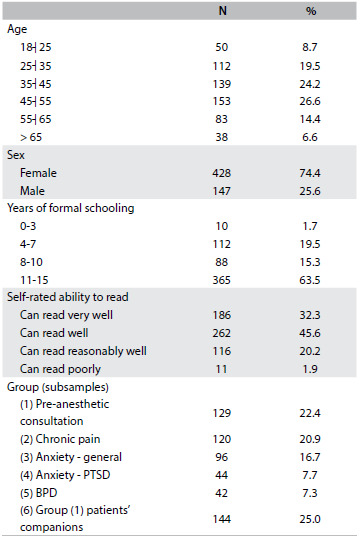
Demographic characteristics of participants in the Connor-Davidson Resilience Scale Brazil (RISC-Br) validation phase

PTSD = post-traumatic stress disorder; BPD = borderline personality disorder.

### Factor structure and reliability

Principal component analysis yielded four components, with eigenvalues of 10.2, 1.5, 1.2 and 1.1. These values accounted for 40.8, 5.8, 4.7 and 4.3% of the total variance, respectively. Oblique rotation was calculated using this four-factor solution, and the resulting factors were named tenacity (items 5, 10-12, 15, 16 and 21-25), adaptability-tolerance (1, 4, 6-8, 14 and 17-19), reliance on support from outside (2, 3 and 13) and intuition (9 and 20) (Table 2).

**Table 2.**
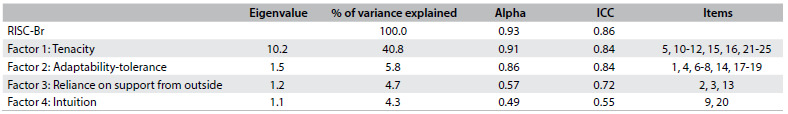
Connor-Davidson Resilience Scale Brazil (RISC-Br) factor structure with items associated with each factor

ICC = intraclass correlation coefficient.

Cronbach’s alpha coefficient was 0.91 for factor 1, 0.86 for factor 2, 0.57 for factor 3, 0.49 for factor 4 and 0.93 for the complete scale. The RISC-Br was completed on a second occasion by 123 participants, after an interval of 7-14 days (median: 10 days). Intraclass correlation coefficient was 0.84 for factors 1 and 2, 0.72 for factor 3, 0.55 for factor 4 and 0.86 for the complete scale (Table 2).

### Construct validity

Spearman correlations were calculated between the RISC-Br and the six subscales of the Brazilian Lipp Stress Symptom Inventory, the Self-Report Questionnaire, the three subscales of the Sheehan Disability Scale and three subscales of the Chronic Pain Grade. Correlations were also calculated between each of these items and each of the four factors of the RISC-Br (Table 3). Significant negative correlations were observed with all but one of the six subscales of the Brazilian Lipp Stress Symptom Inventory, with the Self-Report Questionnaire and with the subscales of the Sheehan Disability Scale. The Spearman correlation coefficients ranged from negative 0.45 to negative 0.26 (P < 0.01). Overall, the correlations were stronger for Factors 1 and 2 and weaker for 3 and 4. Stronger correlations were observed with the psychological than with the physical dimensions of stress symptoms of the Brazilian Lipp Stress Symptom Inventory. Among the three dimensions of the Sheehan Disability Scale, social impairment showed the strongest correlation with the RISC-Br. No correlation was found between the RISC-Br (or its factors) and the dimension of psychological stress symptoms over the last 24 hours in the Brazilian Lipp Stress Symptom Inventory. Among the dimensions of the Chronic Pain Grade, there were significant, although modest, negative correlations between pain intensity and Factor 3, and between pain-related disability and Factor 2 (-0.19 in both cases; P < 0.05).

**Table 3.**
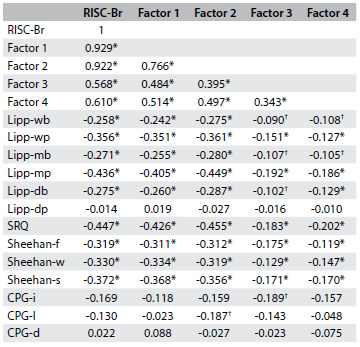
Spearman correlations between the Connor-Davidson Resilience Scale Brazil (RISC-Br), its factors and the external comparison variables

*P < 0.01; ^†^P < 0.05. wb = body symptoms of last week; wp = psychological symptoms of last week; mb = body symptoms of last month; mp = psychological symptoms of last month; db = body symptoms of last day; dp = psychological symptoms of last day; SRQ = Self-Reporting Questionnaire; Sheehan-f = symptoms affect family relations; Sheehan-w = symptoms affect work activities; Sheehan-s = symptoms affect social/leisure activities; CPG-i = pain intensity; CPG-l = activity limitation due to pain; CPG-d = days of limitation.

## DISCUSSION

This paper reports on the cross-cultural adaptation and validation of a Brazilian Portuguese version of the CD-RISC, using selected clinical samples. The RISC-Br showed adequate reliability and validity. A four-factor solution seemed to fit well with the theoretical framework of resilience, and significant correlations with comparison criteria were observed.

Psychometric comparisons between versions of the CD-RISC across cultures should be made cautiously. Beyond the cultural differences, there have been discrepancies in the rotation method (orthogonal or oblique), ages of participants, strategies for questionnaire delivery (from internet-based data-gathering to personal one-to-one interviews) and sources of the samples (population-based, clinical samples, subgroups affected by a specific catastrophic event and so forth).

Differing from the original CD-RISC (which was presented with five factors and varimax rotation), a four-factor solution emerged from the RISC-Br, in accordance with Kaiser’s rule, using either varimax or oblimin rotation. We preferred to analyze the results from oblique rotation, since the domains of the resilience construct were expected to relate to each other.^31^ Furthermore, since the factor structure of the CD-RISC was studied in a community-based sample and that of the RISC-Br in a clinical sample, strict comparison may not be appropriate. Indeed, some investigators have challenged the five-factor solution of the original scale. Campbell-Sills and Stein reported that a four-factor solution was the best fit, in testing the scale using two samples of American undergraduates (around 500 students in each sample). One of these four factors contained items with disparate themes (social support and purpose in life), which led the authors to attempt to refine the scale through dropping several of its items.^18^ In the Turkish validation study, even though five factors were identified, the author reported that the item-factor loadings were dissimilar from those of the original scale.^19^ Furthermore, from the validation studies in China, a three-factor structure emerged from an adult sample,^26^ and was confirmed using adolescents.^24^ A study on South African adolescents also failed to confirm the original five-factor structure of the CD-RISC.^22^

This four-factor solution for the RISC-Br seems to have discarded the spirituality domain of the original scale (which was its fifth factor). The two items that were assumed to relate to spirituality in the original scale (item 3, “Fate or God can help”; and item 9, “Good or bad, most things happen for a reason”) loaded differently but very coherently in the RISC-Br. The former loaded most strongly in the factor of reliance on support from outside, which also harbored item 2 (“I’ve a secure relationship that helps me”) and item 13 (“In times of stress I know where to turn for help”). It is likely that, whether from God or from an acquaintance in the neighborhood, these two items resonated as indistinguishable forms of help from outside in the context of the present sample. Item 9 loaded most strongly in the factor of intuition, where item 20 was also placed (“sometimes you have to act on a hunch, without knowing why”). In these two items of factor 4, there is an intuitive feeling of safeness despite uncertainty. It is noteworthy that in the original study, both items (3 and 9) of the fifth factor (“influences of spirituality”) were considered to be somewhat problematic because they displayed cross-factor loadings and low item-total score correlations.^15^ The same was observed in an Australian study^32^ and among the Chinese population (in this last case, possibly attributable to differences in religious beliefs).^24^

The alpha coefficient of 0.93 that was obtained for the RISC-Br demonstrates that it had good internal consistency, although there is evidence of a certain degree of content redundancy. Redundancy across the scale items has also been noted by authors from other cultural contexts. The two core factors of tenacity and adaptability-tolerance exhibit excellent alpha coefficients, while the modest coefficients of the factors of reliance on external support and intuition can be attributed to the subscale shortness (three and two items respectively). The adequate intraclass correlation coefficients indicated that there was good temporal stability both for the entire RISC-Br and for its subscales.

As expected, the resilience scores correlated negatively with the Self-Report Questionnaire, the Sheehan Disability Scale and the majority of the dimensions of the Brazilian Lipp Stress Symptom Inventory. The lack of correlation between the RISC-Br and the dimension of psychological symptoms over the last 24 hours in the Brazilian Lipp Stress Symptom Inventory can be attributed to the fact that this dimension only comprises three items, which had antagonistic values in relation to the items of the other dimensions. These three items invoke “positive” feelings (“sudden urge to start new projects; excitement; increased motivation”) instead of “negative” distressing symptoms (“dry mouth; dizziness; tiredness”).

This study failed to demonstrate a consistent correlation between chronic pain and resilience, with only two weak correlations arising from two factors of the RISC-Br and two dimensions of the Chronic Pain Grade. Nevertheless, the Chronic Pain Grade showed appropriate psychometrics in its validation study.^30^ It is reasonable to hypothesize that in our sample of chronic pain outpatients, a response artifact may have biased the participants’ answers towards endorsing high levels of symptoms, regardless of their inner resilience, since this would assure them of continuity of care in the public specialized pain clinic. In the Chronic Pain Grade validation study, data on chronic pain was collected from the community.

This study did not aim to test hypotheses. At best, some hypotheses arose. Many authors indicated that personal resilience was a predictor of mental health, and that low resilience was associated with several psychiatric conditions (particularly anxiety disorders).^33^^,^^34^^,^^35^ Within our subsamples, psychiatric patients indeed presented significantly lower resilience scores than those of non-psychiatric patients. There are many recent studies in the psychiatric literature regarding the resilience of post-traumatic stress disorder patients,^5^ and (to our knowledge) none on the resilience of borderline patients. Borderline patients also need to become a paradigmatic source of information regarding the development of personal resilience.

This study presents limitations. First, it was not a population-based study. The absence of a sample from the community precludes any inference about the resilience of Brazilian general population. Second, the psychometrics of two factors (social support and intuition) did not reach good levels. This may have occurred because of the paucity of items devoted to these domains. In this preliminary appraisal on how the RISC-Br would perform within specific clinical samples, we intended to explore its original structure. In further research, confirmatory factor analysis will provide scale refinement, probably through dropping some items. Third, no rigid criteria for recruiting participants were adopted. Nevertheless, the study subsamples were all derived from the same population (clients of the same hospital), which may, to some degree, have restricted the influence of selection bias.

## CONCLUSION

The objective of making an instrument available for measuring personal resilience in Brazil was attained. The RISC-Br showed adequate reliability, temporal stability and construct validity when tested in clinical settings on adult psychiatric and non-psychiatric patients. In the Brazilian version, the 25 scale items clustered within four factors, but the comprehensibility of the factors within a conceptual framework of resilience seems to have been maximized in accordance with the Brazilian cultural context.
